# Antimicrobial Constituents of the Leaves of *Mikania micrantha* H. B. K

**DOI:** 10.1371/journal.pone.0076725

**Published:** 2013-10-02

**Authors:** Yan Li, Jun Li, Yuan Li, Xia-xia Wang, Ao-cheng Cao

**Affiliations:** 1 Institute of Plant Protection, Chinese Academy of Agricultural Sciences, Beijing, P. R. China; 2 College of Resources and Environment, South China Agricultural University, Guangzhou, Guangdong, P. R. China; 3 Institute of Animal Sciences, Anhui Academy of Agricultural Sciences, Hefei, P. R. China; George Mason University, United States of America

## Abstract

**Background:**

To isolate plant-derived compounds with antimicrobial activity from the leaves of *Mikania micrantha*, to determine the compounds configuration, and to evaluate their antimicrobial activity against eight plant pathogenic fungi (*Exserohilum turcicum*, *Colletotrichum lagenarium*, *Pseudoperonispora cubensis, Botrytis cirerea*, *Rhizoctonia solani*, *Phytophthora parasitica*, *Fusarium solani,* and *Pythium aphanidermatum*,) and four plant pathogenic bacteria (gram negative bacteria: *Ralstonia dolaanacearum*, *Xanthomonas oryzae* pv. *Oryzae*, *Xanthomonas Campestris pv. Vesicatoria*, and *Xanthomonas campestris pv. Citri*), and four bacteria (gram positive bacteria: *Staphyloccocus aureus, Bacillus subtilis, Micrococcus luteus,* and *Bacillus cereus*).

**Methods and Results:**

Antimicrobial constituents of the leaves of *M. micrantha* were isolated using bioactivity- guided fractionation. The antifungal activity of the isolated compounds was evaluated by the inhibit hypha growth method and inhibit spore germination method. Characterization of antibacterial activity was carried out using the minimum inhibitory concentrations (MICs) and the minimum bactericidal concentrations (MBCs). MIC and MBC were determined by the broth microdilution method. Six compounds – deoxymikanolide, scandenolide, dihydroscandenolide, mikanolide, dihydromikanolide, and m - methoxy benzoic acid – have been isolated from leaves of *Mikania micrantha* H. B. K. Deoxymikanolide, scandenolide, and dihydroscandenolide were new compounds. The result of bioassay showed that all of isolated compounds were effective against tested strains and deoxymikanolide showed the strongest activity.

**Conclusions and Significance:**

The leaves of *M. micrantha* may be a promising source in the search for new antimicrobial drugs due to its efficacy and the broadest range. Meanwhile, adverse impact of *M. micrantha* will be eliminated.

## Introduction

*Mikania micrantha* H. B. K. (Asteraceae), commonly known as mile-a-minute weed, is an extremely fast-growing, perennial creeping weed. Its native distribution is in Central and South America. It has been present in south China since the 1980s and has caused adverse impact on agricultural production in the established area [1]. So, the weed has been listed as one of the world's 100 worst invasive alien species by Invasive Species Specialist Group of IUCN [2]. The leaves of *M. micrantha*, commonly known as ‘guaco’, are used to make a poultice for snake bites and scorpion sting, decoction of the leaves is used to bathe rashes, and skin itches [3]. In Jamaica, its most popular uses are for wound dressings and promote the healing of sores as folk medicine [4]. In addition, it has been attracted the attention of natural products chemists because of its antibacterial, antitumor, cytotoxic, analgesic, inflammatory, antiproliferative, and phytotoxic activities [5]–[13]. By consulting literatures, sterols [14], [15], diterpenes [15], polyphenols [16], flavonoids [17]–[19], and sesquiterpene lactones [4], [14], [20] have been isolated from *M. micrantha*. However, the antimicrobial constituents were ambiguous, though mikanolide and dihydromikanolide as antimicrobial ingredient of *M. micrantha* were reported. With the aim of identifying those potentially additional and bioactive natural products of this plant, a phytochemical study has been carried out bioactivity-guided fractionation and resulted in the isolation of three new sesquiterpene lactones, named deoxymikanolide, scandenolide, and dihydroscandenolide (**1**–**3**), together with three known compounds (**4**–**6**). Compound **6** was named as m - methoxy benzoic acid, which was the first reported occurrence from this plant. Herein, the isolation and structural elucidation of these compounds, as well as their antimicrobial activities against eight bacteria and eight fungi, were described. This study provides first comprehensive description about the antimicrobial constituent and antimicrobial activities in an attempt to improve the understanding of the practicality of *M. micrantha*.

## Materials and Methods

### Materials

#### Plant material

The leaves of M. micrantha were collected in April 2011, in Dongguan, Guangdong Province of China. The sample was identified by Professor Xiao-yi Wei. An authenticated voucher specimen (No. 020701) was deposited at the herbarium of South China Institute of Botany, Chinese Academy of Sciences, Guangzhou, and People's Republic of China. We state clearly that no specific permissions were required for these locations, because these locations are uncultivated land. We confirm that the field studies did not involve endangered or protected species.

#### Fungal Strains

Eight reference fungi, Exserohilum turcicum, Colletotrichum lagenarium, Pseudoperonispora cubensis, Botrytis cirerea, Rhizoctonia solani, Phytophthora parasitica, Fusarium solani, and *Pythium aphanidermatum*, were used during the study. The tested strains were obtained from Institute of Plant Protection, Chinese Academy of Agricultural Sciences China. The fungi were incubated for 72 h at 28°C in Potato Dextrose Agar (PDA) and stored in plate at 4°C.

#### Bacterial Strains

Eight reference bacteria, Staphyloccocus aureus, Bacillus subtilis, Micrococcus luteus, Bacillus cereus, Ralstonia dolaanacearum, Xanthomonas oryzae pv. Oryzae, Xanthomonas Campestris pv. Vesicatoria, and Xanthomonas campestris pv. citri were used during the study. The tested strains were obtained from the Institute of Plant Protection, Chinese Academy of Agricultural Sciences China. The tested strains cultured in beef extract peptone agar medium (PBA) at 27°C and stored in nutrient agar slants at 4°C.

### Extraction and isolation

The air-dried leaves powder of *M. micrantha* (2.0 kg) was ultrasonic extracted with chloroform (6L×3) at ambient temperature. Actived carbon was added in the extract for decolorizing, then filtered in order to get rid of actived carbon and undissolved substance. The extract was concentrated in vacuo to give 122.60 g of the crude residue (yield 6.13%). The crude residue (120 g) was subjected to chromatography on silica gel (100–200 mesh) eluting successively with gradient carbon tetrachloride – acetone – methanol (35∶2∶1, 35∶5∶1, 35∶5∶2 35∶6∶2, 35∶6∶3, 35∶6∶4, 35∶6∶5, v/v) to give fractions A-1 to A-30. A-11 (1.8434 g) was rechromatographed on a silica gel column eluted with chloroform: ethyl acetate (4∶1 v/v) followed by crystallization from chloroform to give compound **1** (0.8310 g 0.04155% yield) and compound **2** (0.3512 g 0.01756% yield). A-14 (1.2306 g) was rechromatographed on a silica gel column eluted with petroleum ether: ethyl acetate (5∶1 v/v) followed by crystallization from chloroform to give compound **3** (0.4432 g 0.02216% yield). A-16 (5.7534 g) was rechromatographed on a silica gel column eluted with petroleum ether: ethyl acetate (1∶1 v/v) followed by crystallization from acetone to give compound **4** (2.7364 g 0.13682% yield). A-17 (2.5618 g) was rechromatographed on a silica gel column eluted with chloroform: ethyl acetate (1∶1 v/v) followed by crystallization from acetone to give compound **5** (0.8041 g 0.040205% yield). A-19 (1.7658 g) was rechromatographed on a silica gel column eluted with petroleum ether: ethyl acetate: acetone (15∶2∶5 v/v/v) followed by crystallization from chloroform to give compound **6** (0.4355 g 0.021775% yield).

### Deoxymikanolide (1)

Needle crystals; mp 180–185°C; [α] +57.5 (*c* 0.4, CH_3_Cl); UV (CH_3_Cl) λ_max_: 281, 276 nm. IR (KBr) γ_max_: 3435, 2924, 2853, 1757, 1658, 1288, 1154, 1044, 464 cm^−1^. For ^1^H and ^13^C NMR spectroscopic data, see Table 1. Positive HRESIMS *m/z* 277.0294 [M+H]^+^ (calc. for C_15_H_16_O_5_H, 277.2895). The Mass spectral data of deoxymikanolide were 277.0294, 269.0351, 253.06253, and 242.28345.

**Table 1:** ^1^H NMR(400 MHz) and ^13^C NMR (300 MHz) spectral data of compound 1–6 (DMSO) (*J* in Hz).

No.	1		2		3		4		5		6	
	^1^NMR	^1^H NMR	^13^C NMR	^1^H NMR	^13^C NMR	^1^H NMR	^13^C NMR	^1^H NMR	^13^C NMR	^1^H NMR	^13^C NMR	^1^H NMR
1	61.16	2.86 d (9.6)	57.41	3.00 d (10.1)	58.41	2.98 d (10.0)	57.15	3.26 s	58.66	3.30 s	141.36	
2	23.19	1.61 m	28.59	1.62 m	29.02	1.72 m	55.25	3.38 d (3.2)	56.08	3.37 d (2.8)	127.43	7.98 m
		1.07 m		2.24 m		2.49 m						
3	21.66	2.78 m	66.13	5.58 s	67.09	5.60 d (1.0)	49.14	4.79 m	51.54	4.02 m	130.69	
		2.43 m										
4	131.55		130.92		131.48		128.85		129.93		131.94	7.75 dd (1.2)
5	150.77	7.77 s	149.00	7.95 s	149.10	7.97 s	150.19	7.61 d (0.9)	151.15	7.56 m	131.13	8.07 t (11.6)
6	82.30	5.47 m	81.99	5.61 d (20.0)	80.72	5.41 s	83.2	5.34 m	83.01	5.45 s	128.85	7.79 dd (3.2, 11.2)
7	49.65	3.46 dd (21.0, 17.1	48.67	3.52 dd (45.7, 41.8)	52.95	4.06 m	50.01	3.06 m	53.50	2.96 td (13.8, 6.9)	167.75	
8	78.08	4.63 m	77.27	4.66 m	77.71	4.63 m	76.48	4.81 m	77.97	4.64 m	52.83	3.92 s
9	43.31	1.86 dd (13.6, 4.3)	41.93	1.91 dd (13.7, 4.1)	42.37	1.96 dd (5.0, 5.0)	42.14	1.88 dd (13.4, 4.6)	43.49	1.86 dd (13.5, 10.9)		
		2.26 dd (28.9, 13.2)		2.17 dd (16.0, 16.0)		2.10 dd (5.0, 5.0)		2.19 dd (13.1, 11.3)		2.07 dd (13.6, 4.2)		
10	57.16		56.50		57.27		57.07		58.83			
11	138.08		136.74		40.80	2.67 m	138.25		42.30	2.98 m		
12	168.51		167.60		176.31		167.88		177.36			
13	122.66	6.23 d (3.5)	122.07	6.25 d (3.5)	13.37	1.29 d (5.0)	121.98	6.22, d (3.5)	14.53	1.26 d (7.0)		
		6.05 d (3.0)		6.08 d (2.9)				5.95 d (3.0)				
14	20.34	1.06 s	19.39	1.09 s	20.21	1.08 d (6.0)	20.90	1.00 s	22.17	0.96 d (9.2)		
15	172.67		169.14		170.30		170.78		172.30			
OAc			169.61		169.83							
			20.43	2.15 s	21.20							

### Scandenolide (2)

Needle crystals; mp 160–165°C; [α] +43.2 (c 0.5, CH_3_Cl); UV (CH_3_Cl) λ_max_: 262, 273 nm. IR (KBr) γ_max_: 3435, 2924, 2853, 1770, 1659, 1369, 1284, 1239, 685 cm^−1^. For ^1^H and ^13^C NMR spectroscopic data, see Table 1. Positive HRESIMS *m/z* 357.2945 [M+Na]^+^ (calcd for C_17_H_18_O_7_Na, 357.3161). The Mass spectral data of scandenolide were 357.2945, 338.3420, 321.0502, 301.1412, 280.2635, 253.0630, and 238.0146.

### Dihydroscandenolide (3)

Needle crystals; mp 270–275°C; [α] +54.3 (*c* 0.1, CH_3_Cl); UV (CH_3_Cl) λ_max_: 257, 288 nm. IR (KBr) γ_max_: 3421, 2921, 1750, 1639, 1235, 1154, 1034, 675 cm^−1^. For ^1^H and ^13^C NMR spectroscopic data, see Table 1. Positive HRESIMS *m/z* 337.0241 [M+H]^+^ (calc. for C_17_H_20_O_7_H, 337.3502). The Mass spectral data of dihydroscandenolide were 337.0241, 321.0514, 305.0736, 286.0263, and 270.0535.

### Single crystal X-ray diffraction analysis of 1, 2, and 3

Upon crystallization from CHCl_3_-EtOAc (1∶1) using the vapor diffusion method, colorless needles of compound **1** were obtained. A suitable crystal was selected and performed on a SuperNova, Dual, and Cu at zero, Atlas diffractometer. The crystal was kept at 100.01(10) K during data collection. Using Olex2 [21], the structure was solved with the XS [22], structure solution program using Direct Methods and refined with the XL [22] refinement package using Least Squares minimisation. Crystal data: C_15_H_16_O_5_, *M* = 276.28, space group monoclinic, P2_1_; unit cell dimensions were determined to be a = 7.1569 (6) Å, α = 90.00°; b = 6.0316 (5) Å, β = 91.177 (9) °; c = 14.6466 (13) Å, γ = 90.00°; V = 632.12 (9) Å^3^, *Z = *2, Dcalc = 1.452 mg/mm^3^, crystal size 0.2×0.1×0.05, μ (Mo Kα) = 0.109 mm^−1^, F(000) = 292.0, *T* = 100.01(10) K, 2359 reflections measured (5.7≤2Θ≤50.04), 1678 unique (*R*_int_ = 0.0684) which were used in all calculations. The final *R*_1_ was 0.0764 (> 2sigma (I)) and *wR*_2_ was 0.3147. In the structure refinements, nonhydrogen atoms were placed on the geometrically ideal positions by the “ride on” method. Hydrogen atoms bonded to oxygen were located by the structure factors with isotropic temperature factors. Crystallographic data for **1** have been deposited at the Cambridge Crystallographic Data Centre (deposition No. CCDC-939627).

Upon crystallization from CHCl_3_ using the vapor diffusion method, colorless needles of compound **2** were obtained. A suitable crystal was selected and performed on a SuperNova, Dual, and Cu at zero, Atlas diffractometer. The crystal was kept at 100.01(10) K during data collection. Using Olex 2 [21], the structure was solved with the Superflip [23] structure solution program using Charge Flipping and refined with the XL [22] refinement package using Least Squares minimisation. Crystal data: C_17_H_18_O_7_, *M* = 334.31, space group monoclinic, P2_1_; unit cell dimensions were determined to be a = 6.2575 (8) Å, α = 90.00°; b = 9.4695 (8) Å, β = 91.897 (10) °; c = 13.3357 (12) Å, γ = 90.00°; V = 789.78 (13) Å^3^, *Z = *2, Dcalc = 1.406 mg/mm^3^, crystal size 0.3×0.2×0.1, μ (Mo Kα) = 0.110 mm^−1^, F(000) = 352.0, *T* = 100.01(10) K, 2749 reflections measured (7.48≤2Θ≤50.02), 2019 unique (*R*_int_ = 0.0384) which were used in all calculations. The final *R*_1_ was 0.0526 (> 2sigma (I)) and *wR*_2_ was 0.1384. In the structure refinements, nonhydrogen atoms were placed on the geometrically ideal positions by the “ride on” method. Hydrogen atoms bonded to oxygen were located by the structure factors with isotropic temperature factors. Crystallographic data for **2** have been deposited at the Cambridge Crystallographic Data Centre (deposition No. CCDC- 922241).

Upon crystallization from CHCl_3_-EtOAc (2∶1) using the vapor diffusion method, colorless needles of compound **3** were obtained. A suitable crystal was selected and performed on a SuperNova, Dual, and Cu at zero, Atlas diffractometer. The crystal was kept at 180.00(10) K during data collection. Using Olex 2 [21], the structure was solved with the Superflip [23] structure solution program using Charge Flipping and refined with the XL [22] refinement package using Least Squares minimisation. Crystal data: C_17_H_20_O_7_, *M* = 336.33, space group monoclinic, P2_1_; unit cell dimensions were determined to be a = 6.2346 (3) Å, α = 90.00°; b = 9.5598 (5) Å, β = 91.687 (5) °; c = 13.4299 (7) Å, γ = 90.00°; V = 800.09 (7) Å^3^, *Z = *2, Dcalc = 1.396 mg/mm^3^, crystal size 0.2×0.2×0.1, μ (Mo Kα) = 0.109 mm^−1^, F(000) = 356.0, *T* = 180.00(10) K, 4879 reflections measured (6.54≤2Θ≤52.04), 2719 unique (*R*_int_ = 0.0221) which were used in all calculations. The final *R*_1_ was 0.0362 (> 2sigma (I)) and *wR*_2_ was 0.0854. In the structure refinements, nonhydrogen atoms were placed on the geometrically ideal positions by the “ride on” method. Hydrogen atoms bonded to oxygen were located by the structure factors with isotropic temperature factors. Crystallographic data for **3** have been deposited at the Cambridge Crystallographic Data Centre (deposition No. CCDC- 939629).

### Antibacterial activity assay

MIC and MBC of the compounds were assessed using the broth microdilution method recommended by the National Committee for Clinical Laboratory Standards [24], [25]. Inoculums of the microorganism were prepared from 24 h Mueller-Hinton Broth (MHB) cultures and suspensions were adjusted with turbidity equivalent to that of a 0.5 McFarland standard. The compounds dissolved in 5% dimethylsulfoxide (DMSO), were first diluted to the highest concentration (2000 mg/L) to be tested, and then serial twofold dilutions were made in a concentration range from 15.625 to 1000.0 mg/L in 5 ml sterile test tubes containing the medium. The 96-well plates were prepared by dispensing into each well 95 µL of nutrient broth and 5 µL of the inoculum. A 100 µL aliquot from the stock solutions of each isolates was added into the first wells. Then, 100 µL from the serial dilutions were transferred into 6 consecutive wells. The last well containing 195 µL of the medium without the compounds and 5 µL of the inoculum on each strip was used as negative control. The final volume in each well was 200 µL. Broth with 5 µL of DMSO was used as blank test. Plates were covered and incubated for 12 h at 37°C. After incubation, the lowest concentration of tested samples, which did not show any visual growth after macroscopic evaluation, was determined as MIC. Using the results of the MIC assay, the concentrations showing complete absence of visual growth of bacteria were identified and 10 mL of each culture broth was transferred on to the agar plates and incubated for the specified time and temperature as mentioned above. The complete absence of growth on the agar surface in the lowest concentration of sample was defined as the MBC. All tests were performed in triplicate.

### Antifungal activity assay

Antifungal activities of the compounds was tested using the inhibit hypha growth method. The compounds were prepared with different concentrations using dimethyl sulfoxide (DMSO) and diluted by Potato Dextrose Agar (PDA) medium as 500, 250, 125, 62.5, and 31.25 mg/L. The PDA mediums mixed with the compounds were dumped respectively into petri dishes (9 cm diameter) as plating. An agar plug of fungal inoculums (6 mm in diameter) was removed from a previous culture of the fungal strains tested and placed upside down in the center of the petri dishes. The same amount of DMSO and distilled water which were used to replace the compounds were added respectively into the PDA mediums as the blank test and negative control. Each treatment was done with three replicates. All of materials were subjected to autoclaving at 121°C for 30 min. The means diameter of fungal colony was measured by criss-cross method with calipers by incubated at 28°C.

### Inhibit spore germination activity assay

The spore suspensions of *Botrytis cinerea, Glomerella cingulata, Exserohilum turcicum, Fusarium solani* were obtained from their respective 10 days old cultures. The spore suspension was adjusted with sterile distilled water to a concentration of approximately 1.0×10^5^ spore/mL. The compounds dissolved in 5% dimethyl sulfoxide (DMSO), and then serial twofold dilutions with the spore suspensions were made in a concentration range from 125.0 to 2000.0 mg/L. 100 µL of the spore suspensions were taken onto the glass slide and incubated at 24±2°C for 24 h. About 200 spores were counted and percentage of spore germination was calculated. The same amount of DMSO and distilled water were used as negative control and blank test. All experiments were conducted in triplicate. All of materials were subjected to autoclaving at 121°C for 30 min.

### Statistical analysis

Data were analyzed using SPSS 10.0 (Chicago, IL, USA). The data were considered statistically significant for P values ≤0.05.

## Results

**Figure 1:**
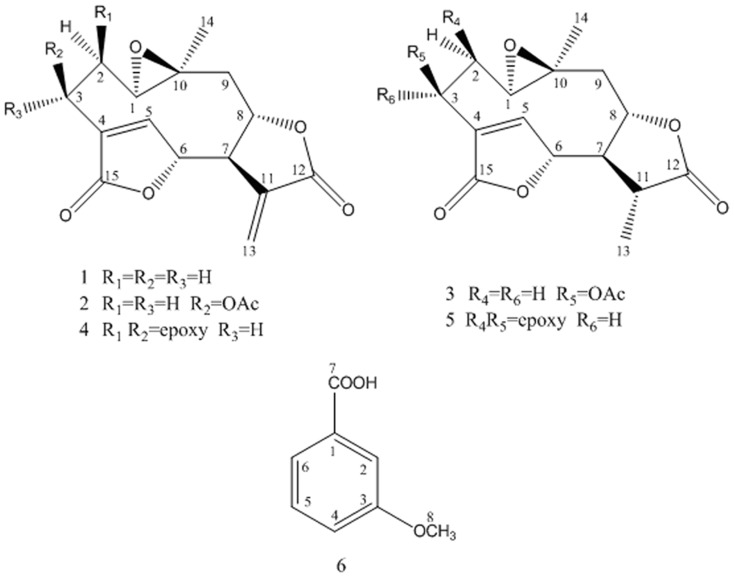
Structures of compounds from the leaves of *Mikania micrantha*. Solid bonds indicate carbon chain. Wedge bonds indicate forwardly chemical bond. Hashed wedged bonds indicate rearwards chemical bond.

Compound **1**–**6** were isolated from the leaves of *M. micrantha* using bioactivity-guided fractionation and their structures were showed in Figure 1.

**Figure 2:**
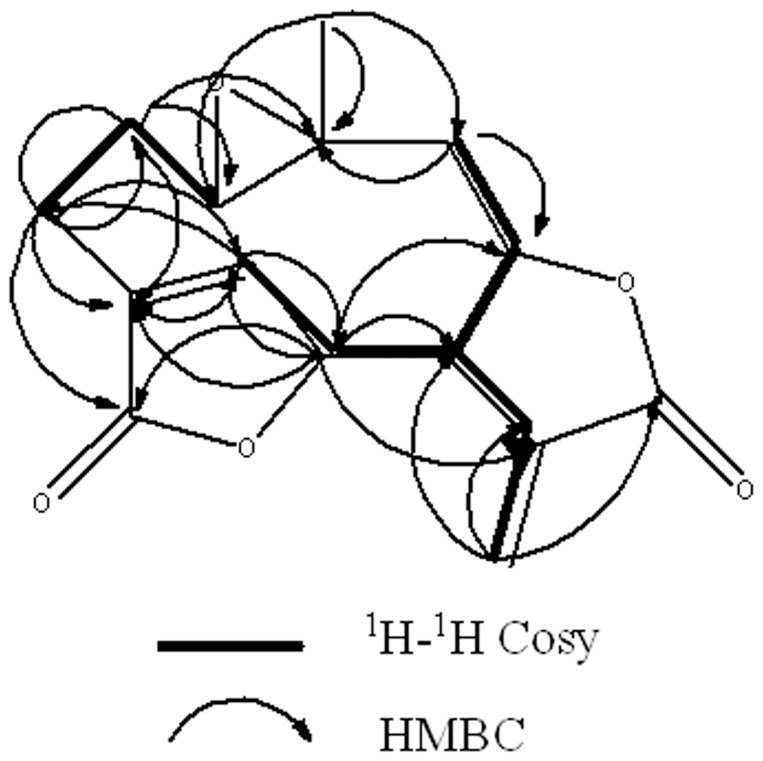
Selected ^1^H-^1^H COSY and HMBC corrections for deoxymikanolide. COSY means correlated spectroscopy and HMBC means (1H-detected) heteronuclear multiple-bond correlation. Bold bond indicate the correlation of hydrogen atoms of adjacent carbon. Arrowed line indicates that hydrogen atom correlate with neighboring carbon (arrow for the carbon atom).

**Figure 3:**
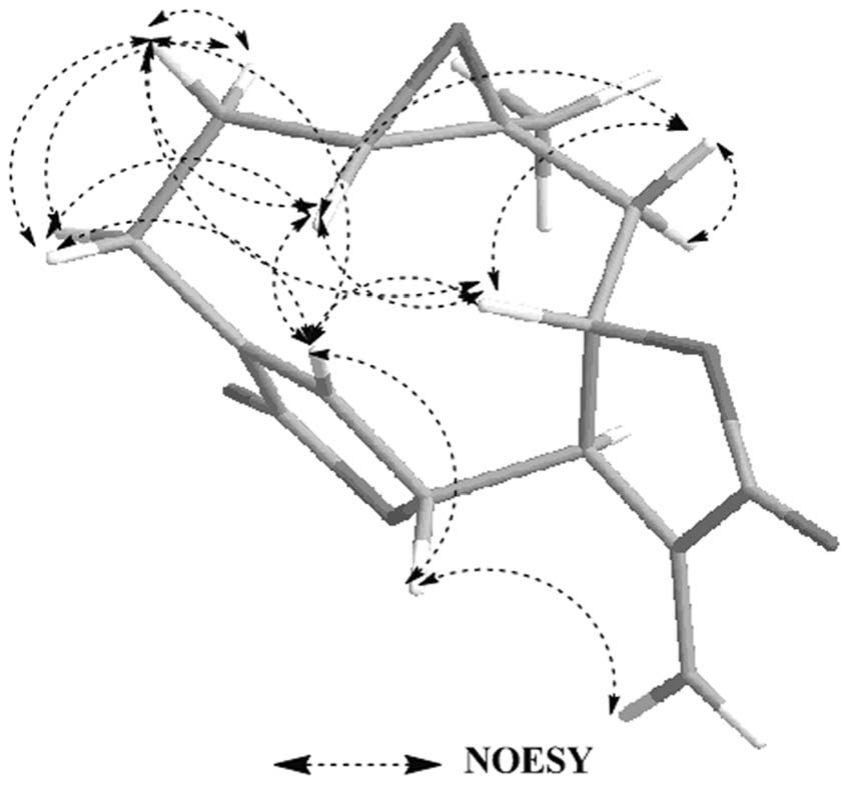
Selected NOESY correlations and relative configuration for deoxymikanolide. NOESY means nuclear Overhauser effect spectroscopy. Dashed bonds indicate the correlation of hydrogen atoms of carbon atoms in two-dimensional space.

**Figure 4:**
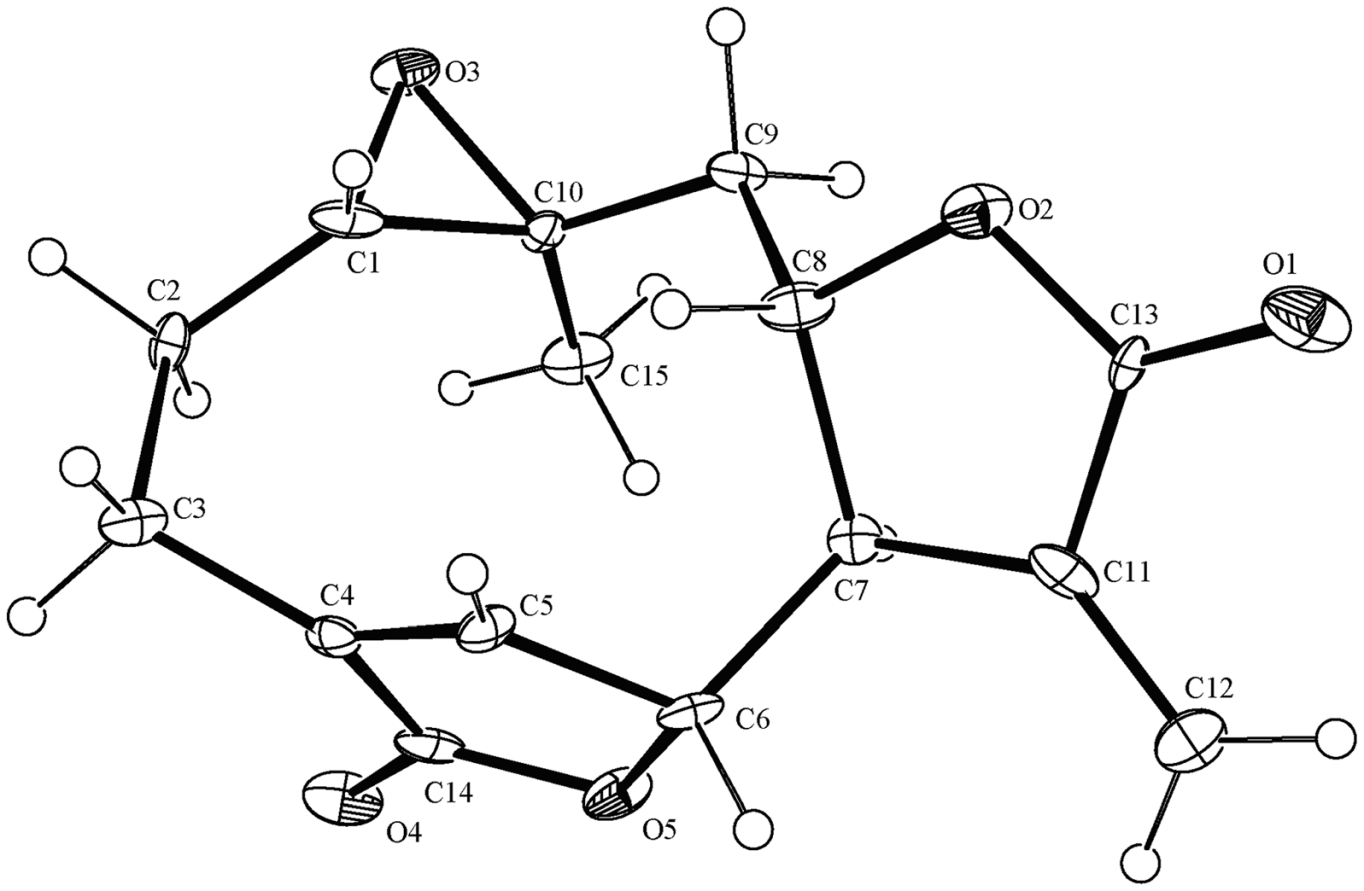
Single-crystal X-ray structure of deoxymikanolide. C letters indicate carbon atoms and the number of C letter indicate carbon chain sequence number. O letters indicate oxygen atoms and the number of O letter indicate oxygen atoms sequence number.

Compound **1** was obtained as colorless needle crystals in chloroform, and gave [M+H]^+^ peak at *m/z* 277.0294 (calc. for C_15_H_16_O_5_H, 277.2895) in HR-ESI-MS, corresponding to the molecular formula C_15_H_16_O_5_, indicating eight degrees of unsaturation. Compared with the spectral data of the sesquiterpene dilactone isolated from this plant, its 1D NMR spectroscopic data were suggestive of the presence of a special guaianolide skeleton (Table 1). In the ^1^H-^1^H COSY experiments, the correlations of H-1 through H-2 to H-3, H-5 through H-6, H-7, H-8, and H-9, to H-10, H-7 to H-13 established two fragments. The HMBC correlations between olefinic proton H-5/C-4, C-6, and C-15 suggested the presence of cyclic olefinic lactone functionality. Moreover, the observed correlation of olefinic group (C-11 and C-13), the ester carbon (C-12), and exocyclic olefinic protons (H-13a and 13b) authenticated the existence of characteristic methylene lactone functionality (Figure 2). On the basis of above data and compared with the spectral data of known sesquiterpenes dilactones isolated from this plant, compound **1** was identified as deoxymikanolide [19], [26]. But its relative stereochemistry have never been reported, which these papers only listed ^13^C and ^1^H NMR spectral data. The relative stereochemistry of **1** was further confirmed by detailed analysis of NOESY spectra (Figure 3) and single-crystal-X-ray diffraction study (Figure 4). In the NOESY spectrum, the correlations of H-1/H-8, and H-6/H-1 indicated that H-1, H-6, and H-8 adopted the same orientation and were arbitrarily designated as the β-orientation. The NOESY correlations of H-14/H-7 revealed that Me-14 and H-7 were α-orientation (Figure 3). Its configuration as established by NOESY spectrum was in good agreement with the X-ray diffraction study. A single crystal of **1** was obtained and subjected to X-ray crystallographic diffraction analysis. The result unambiguously confirmed the structure of **1** and the relative stereochemistry of **1** was determined to be 1R, 6R, 7R, 8S, and 10S. Consequently, compound **1** was elucidated as 1,10- epoxy-6,8-dihydroxy-11-vinylgermacr-4-ene12, 14-di-γ- lactone and 7,10a- dimethyl-1a,1b,2a,6a,7,9a,10,10a-octahydro-4*H*-6,3-methenofuro[3,2-c] bisoxireno[*f,h*] oxacycloundecin-4,8(6*H*)-dione, trivially named deoxymikanolide (Figure 1).

**Figure 5:**
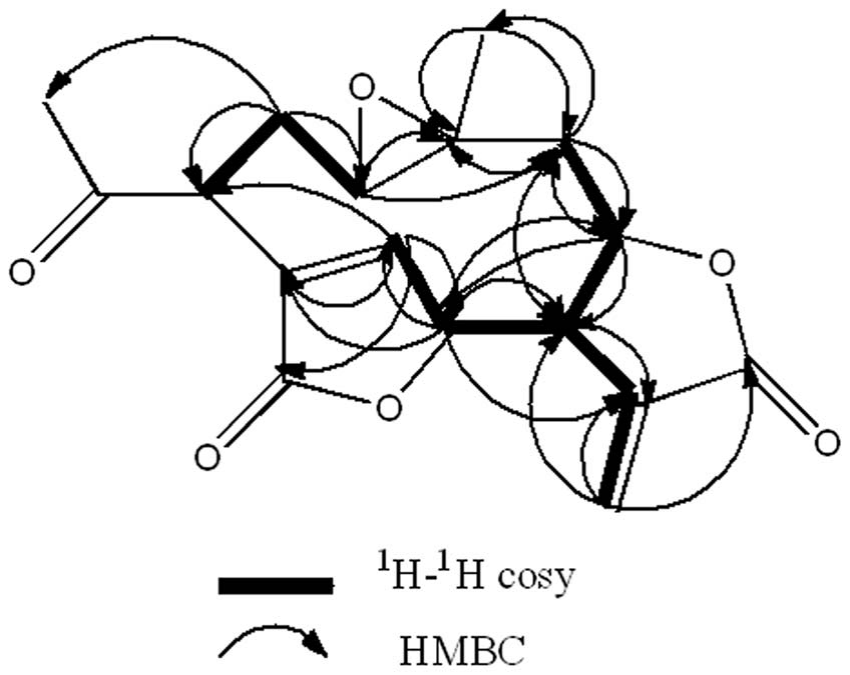
Selected ^1^H-^1^H COSY and HMBC corrections for scandenolide. As shown in figure 2.

**Figure 6:**
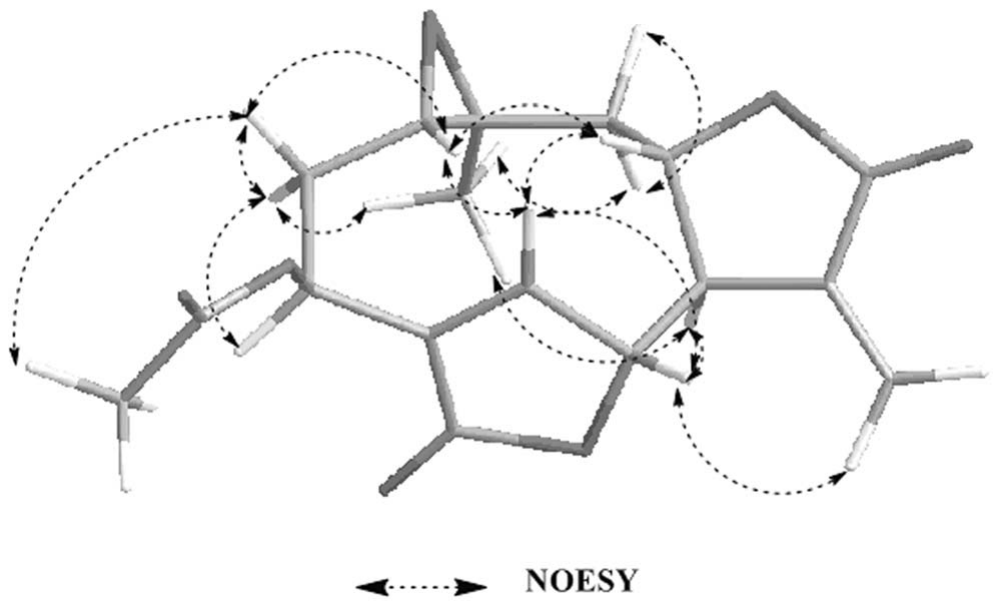
Selected NOESY correlations and relative configuration for scandenolide. As shown in figure 3.

**Figure 7:**
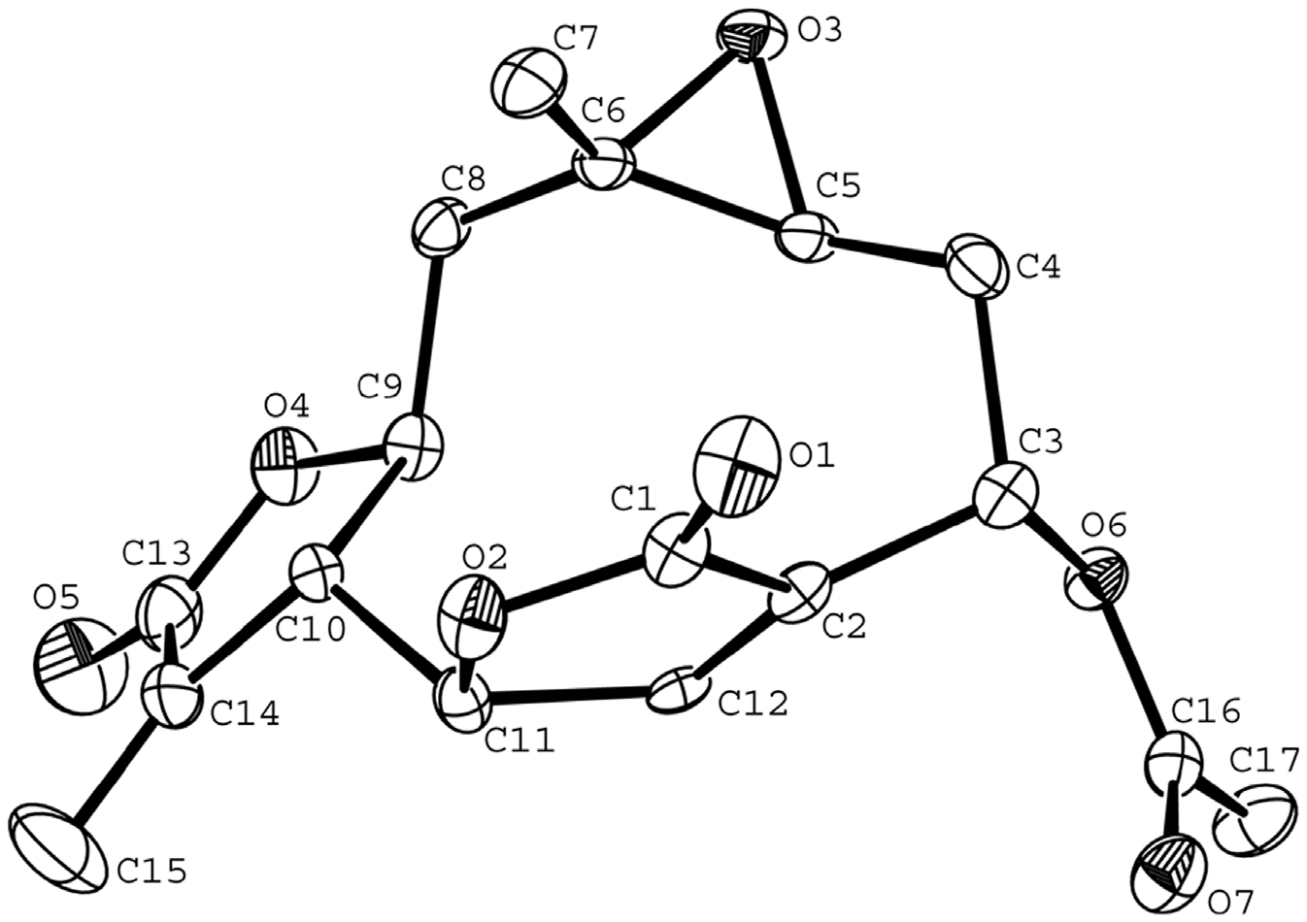
Single-crystal X-ray structure of scandenolide. As shown in figure 4.

Compound **2** was obtained as colorless needle crystals in chloroform, and gave [M+Na]^+^ peak at *m/z* 357.2945 (calc. for C_17_H_18_O_7_Na, 357.3161) in HR-ESI-MS, corresponding to the molecular formula C_17_H_18_O_7_, indicating nine degrees of unsaturation. Just as compound **1**, its 1D NMR spectroscopic data were suggestive of the presence of a special guaianolide skeleton too (Table 1). ^1^H-^1^H COSY and HMBC data of **2** indicated that the structure of **2** familiar with the structure of **1** (Figure 5). However, the ^13^C NMR spectrum showed three carbon signals which were resolved as carbons at δ_C_ 167.6, 169.1, and 169.1, for three acyls. Compared with the spectral data of known sesquiterpenes dilactones isolated from this plant, **2** was identified as scandenolide [19], [26], [27]. Just as compound **1**, no literature detailed the relative stereochemistry of scandenolide too, which these papers only listed ^13^C and ^1^H NMR data. The relative stereochemistry of **2** was further confirmed by detailed analysis of NOESY spectra (Figure 6) and a single-crystal-X-ray diffraction study (Figure 7). Based on the NOESY correlations of H-1/H-2_β_ and H-2_β_/OAc, H-2α/H-14 and H-2α/H-3, OAc as β-orientation have been derived (Figure 6). Configuration of the others chiral carbon atoms as established by NOESY spectrum was in good agreement with the single-crystal-X-ray diffraction study. So, the relative stereochemistry of **2** was determined to be 1R, 3S, 6R, 7R, 8S, and 10S. Consequently, compound **2** was elucidated as 3-ethanoyl-1,10-epoxy-6,8-dihydroxy-11-vinylgermacr-4-ene12,14-di-γ- lactone and 7,10a-dim-ethyl-1a,1b,2a,6a,7,9a,10,10a-octahydro-4*H*-6,3-methenofuro[3,2-c] bisoxireno[*f,h*]oxacyclounde-cin-4,8(6*H*)-dione, trivially named scandenolide (Figure 1).

**Figure 8:**
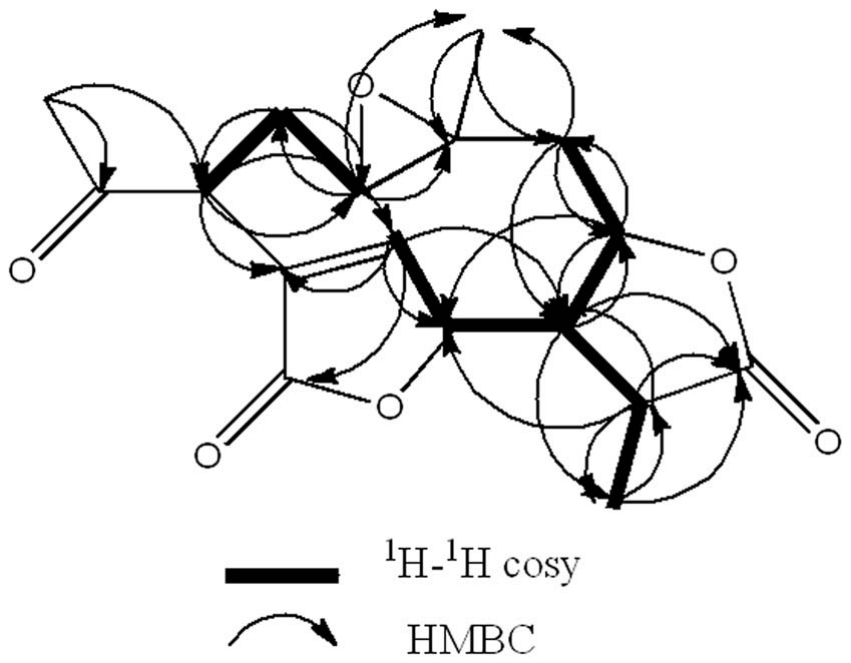
Selected ^1^H-^1^H COSY and HMBC corrections for dihydroscandenolide. As shown in figure 2.

**Figure 9:**
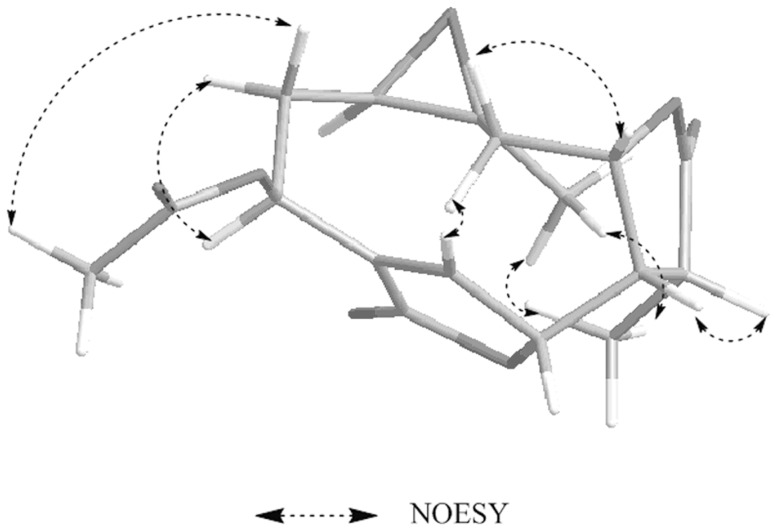
Selected NOESY correlations and relative configuration for dihydroscandenolide. As shown in figure 3.

**Figure 10:**
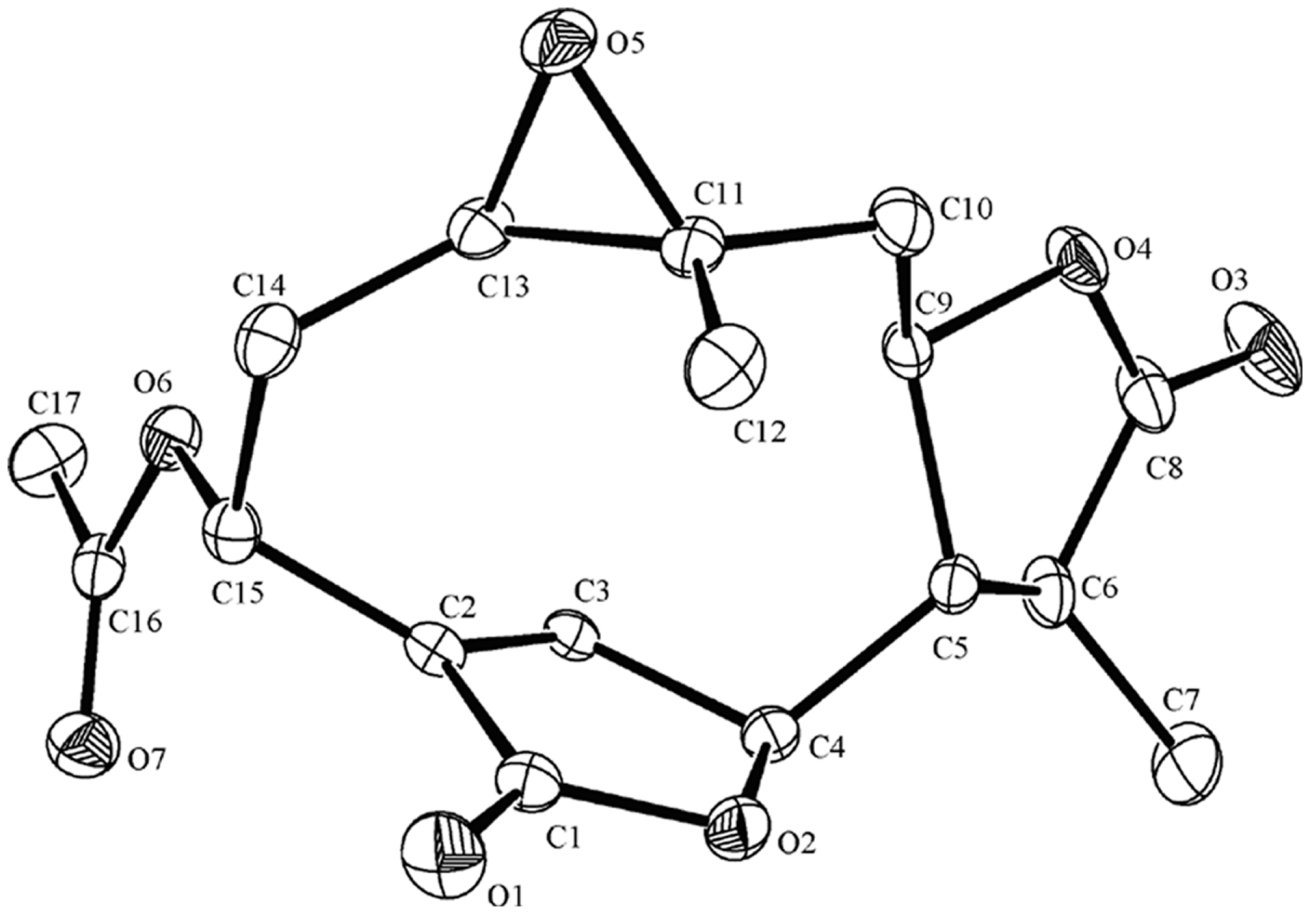
Single-crystal X-ray structure of dihydroscandenolide. As shown in figure 4.

Compound **3** was obtained as colorless needle crystals in chloroform, and gave [M+H]^+^ peak at *m/z* 337.0241 (calc. for C_17_H_20_O_7_H, 337.3502) in HR-ESI-MS, corresponding to the molecular formula C_17_H_18_O_7_, indicating eight degrees of unsaturation. Just as compound **1** and **2**, special guaianolide skeleton was shown obviously according to its 1D NMR spectroscopic data (Table 1). Though, the ^13^C-NMR spectrum showed two carbon signals which were resolved as olefinic carbons at δ_C_ 131.4, and 149.1. Compared with the spectral data of known sesquiterpenes dilactones isolated from this plant, **3** was identified as dihydroscandenolide [19], [26]. Just as compound **1** and **2**, the relative stereochemistry of dihydroscandenolide was not clear. The relative stereochemistry of **3** was further confirmed by detailed analysis of ^1^H-^1^H COSY, HMBC data (Figure 8), and NOESY spectra (Figure 9) and a single-crystal-X-ray diffraction study (Figure 10). In the NOESY spectrum, the correlations of H-13/H-14 revealed that Me-14 and C-13 were α-orientation (Figure 9). Its configuration as established by NOESY spectrum was in good agreement with the X-ray diffraction study. The conformation of **3** established by NOESY spectrum was in good agreement with the single- crystal-X-ray diffraction study and the relative stereochemistry of **3** was determined to be 1R, 3S, 6R, 7R, 8S, 10S, and 11R. Consequently, compound **3** was elucidated as (3R,3aR,4R,8S,9aR,10aS,11aS)-3,10a-dimethyl-2,6-dioxo-3,3a,4,6, 8,9,9a,10a,11,11a-decahydro-2H-4,7-(metheno)furo[3,2-c]oxireno[2,3-f][1]oxacycloundecin-8-yl acetate, trivially named dihydroscandenolide (Figure 1).

Compounds **4**-**6** (Figure 1) were, respectively, identified as mikanolide, dihydromikanolide [19], and m - methoxy benzoic acid by comparison of their spectral data with those reported in the literature. ^1^H and ^13^C NMR spectral data of these compounds were shown in Table 1. Particularly, this is the first reported occurrence of m - methoxy benzoic acid from this plant.

All 6 compounds were subjected to quantitative bioassays for antimicrobial activity. DMSO used at 2.5% (v/v) showed no antimicrobial activity against all tested microorganisms (data not shown). As shown in Table 2, 3, and 4, all isolates were found to possess antimicrobial activity. Among these compounds, **1** was shown to possess significantly more antibacterial activity compared with the others against tested bacteria grown in suspension; the MIC values for tested strains (n = 8) ranged from 62.5 to 125 and the MBC values ranged from 125 to 250 (Table 2). The others were showed moderate activity against all tested bacteria. By the inhibit hypha growth method, it was seen that EC_50_ values of 6 isolated for tested fungi (n = 4) ranged from 82.40 to 330.10 mg/L in different times (Table 3). It is observed that antifungal susceptibility of 6 isolated was not significantly different. The results of inhibit spore germination assay showed that **1** was observed the highest inhibition activity; IC_50_ values ranged from 21.44 to 53.18 mg/L (Table 4). Compound **6** was weakest in activity.

**Table 2:** Inhibitory effects of compound 1-6 isolated from leaves of *M. micrantha* against tested bacteria strains.

Compounds	1		2		3		4		5		6	
	MIC(mg·L^−1^)	MBC(mg·L^−1^)	MIC(mg·L^−1^)	MBC(mg·L^−1^)	MIC(mg·L^−1^)	MBC(mg·L^−1^)	MIC(mg·L^−1^)	MBC(mg·L^−1^)	MIC(mg·L^−1^)	MBC(mg·L^−1^)	MIC(mg·L^−1^)	MBC(mg·L^−1^)
*Staphyloccocus aureus*	62.5	125	125	250	250	500	500	1000	250	500	500	1000
*Bacillus subtilis*	62.5	250	125	250	125	250	250	500	500	1000	500	1000
*Micrococcus luteus*	125	250	125	250	250	500	250	500	500	1000	500	1000
*Bacillus cereus*	125	250	125	250	125	250	500	1000	250	500	500	1000
*Ralstonia dolaanacearum*	62.5	125	125	250	125	250	125	250	125	250	250	500
*Xanthomonas oryzae* pv. *Oryzae*	125	250	250	500	250	500	250	500	250	500	500	1000
*Xanthomonas Campestris* pv. *Vesicatoria*	62.5	125	125	250	125	250	125	250	250	500	500	1000
*Xanthomonas campestris* pv. *citri*	62.5	125	125	250	250	500	125	250	500	500	500	1000

**Table 3:** Antifungal activity of compound 1–6 against tested fungi strains.

Tested Fungus Strains	Time (h)	EC_50_ (mg·L^1^)					
		1	2	3	4	5	6
*Fusarium solani*	48	82.40	101.42	100.23	107.91	100.23	95.28
	72	97.68	126.27	113.07	110.25	113.07	116.15
	96	116.74	144.51	121.38	117.96	121.38	122.96
*Rhizoctonia solani*	48	109.59	117.60	132.29	138.35	132.29	135.04
	72	157.44	169.32	174.17	197.16	174.17	194.32
	96	263.64	282.98	290.77	329.19	290.77	310.00
*Pythium aphanidermatum*	48	114.43	153.20	212.90	205.88	212.90	217.63
	72	129.12	222.83	279.25	254.78	279.25	273.80
	96	167.26	254.34	347.33	288.15	347.33	330.10
*Phytophthora parasitica*	48	116.17	161.10	145.03	149.22	145.03	249.88
	72	125.53	169.88	166.72	159.47	166.72	278.10
	96	156.73	166.53	205.12	153.16	205.12	316.41

**Table 4:** Inhibition of spore germination activity of compound 1–6 against tested fungi strains.

Tested Fungus Strains	IC_50_ (mg·L^−1^)					
	1	2	3	4	5	6
*Exserohilum turcicum*	21.44	86.99	93.42	80.41	76.04	255.37
*Botrytis cirerea*	36.53	113.08	77.55	86.40	62.33	123.51
*Pseudoperonispora cubensis*	53.18	75.18	79.38	132.19	67.78	211.24
*Colletotrichum lagenarium*	40.56	110.97	64.58	80.36	81.34	167.68

## Discussion

In recent decade, problems of multidrug resistant microbes have reached an alarming level in around the world. These pose a serious challenge to the scientific community hence emphasis has been laid on the development of new antimicrobial agents [28], [29]. One possible strategy is the rational localization of bioactive products from folk medicines, with the hope that systematic screening of these will result in the discovery of novel effective compounds with potent and useful activities against microbes. Now, there is an ever-increasing demand for plant-based therapeutics in both developing and developed countries due to a growing recognition that they are natural products, non-narcotic and, in most cases, easily available at affordable prices; they also have no side effects.

In China, since its introduction in the late 1980s and the early 1990s, *M. micrantha* has drastically infested Guangdong Province and caused significant damage to forests, farmlands, and orchards; and led to great loss of native species diversity, significant decline of microbial communities and food web stability; and even altered mineral cycling [30]. Though, *M. micrantha* was used in the traditional pharmacopoeia in some countries. So, the aims of this study not only develop to new antimicrobial agents, but also providing theoretic basis for the rational exploiting and controlling of *M. micrantha*.

Early reports on *M. micrantha* revealed its antimicrobial action; mikanolide and dihydromikanolide were major antimicrobial activity constituents [4]. In the present study, antimicrobial activity compounds were further isolated and the 6 compounds were obtained using bioactivity-guided fractionation, including 3 new sesquiterpene dilactones - deoxymikanolide, scandenolide, and dihydroscandenolide, and 3 known isolates - mikanolide, dihydromikanolide, and m - methoxy benzoic acid. Structure elucidation of 3 new sesquiterpene dilactones were determinated according to 1D, 2D NMR, IR, and single-crystal-X-ray diffraction study. In order to reveal effective substances for bioactivities *in vitro*, 6 isolates were tested for antibacterial activities against four gram-negative and four gram-positive bacteria by the broth microdilution methods. The obtained MIC and MBC values ranged from 62.5 to 1000 mg/L, especially, deoxymikanolide possessed the highest antimicrobial activity; the others only demonstrated moderate antimicrobial activities. Antifungal activities of 6 isolates were described against eight plant pathogens fungi. The results showed that EC_50_ values ranged from 82.40 to 330.10 mg/L in different times by the inhibit hypha growth method and IC_50_ values ranged from 21.44 to 255.37 mg/L by inhibit spore germination method. This study showed that antimicrobial constituents of *M. micrantha* were separated. In addition, structures of five compounds possess special guaianolide skeleton, familiar structure of the compounds were reported in *Mikania* species [8], [31]–[33]. So, the skeleton as parent structure should synthesis and structure-function relationship should further study. Occurrences of dilactone in this special guaianolide skeleton suggest that the problem of water-solubility of these active compounds will been solved by hydrolysis reaction, but bioactivity of the compounds will been tested in future experiment.

The results further demonstrated the broadest range of antimicrobial properties of *M. micrantha*. Considering the broadest range of antimicrobial properties, this would suggest that *M. micrantha* might be useful as a broad-spectrum antimicrobial. The mechanism of action will be done in further study on the basis of *in vitro* experimentation for development of new products. The isolation and bioactivity results will provide scientific foundation for rational development and utilization of this plant. If possible from collected specifically for controlling pathogen agent use, in order to manage indirectly and reduce adverse impact about *M. micrantha*.
